# A perspective on the scope of videoconferencing-based telemedicine in respiratory diseases outpatient clinic

**DOI:** 10.5578/tt.20239602

**Published:** 2023-12-04

**Authors:** Nazlı ÇETİN, Pınar BOSTAN, Göksel ALTINIŞIK

**Affiliations:** 1 Department of Pulmonary Diseases, Pamukkale University Faculty of Medicine, Denizli, Türkiye; 2 Department of Pulmonary Diseases, İstinye University Faculty of Medicine, İstanbul, Türkiye

## Abstract

**ABSTRACT**

**
A perspective on the scope of videoconferencing-based telemedicine in respiratory diseases outpatient clinic
**

**Introduction:** *Telemedicine is rapidly expanding across various fields world- wide. While it finds application in respiratory diseases due to the imperative need for protection against the risk of transmission and the close monitoring of patients with chronic diseases, there is a scarcity of publications detailing telemedicine experiences in respiratory diseases. This study aims to retrospec- tively evaluate the prospective management of patients with respiratory dis- eases through videoconference-based telemedicine, intending to establish a foundation for its judicious application in pulmonology cases.
*

**Materials and Methods:** *In this descriptive study, anonymized medical records of all 478 patients assessed via videoconference-based telemedicine over an eight-month period from June 2020 to February 2021 were reviewed. The analysis included demographic characteristics, disease history, attendance methods, the necessity for in-person physical examination after the initial videoconference (VC) session, the inclusion of investigations, pre-diagnosis, diagnosis processes, follow-up period, and outcomes. Follow-up data for all patients included in the study were reviewed at the end of June 2021.
*

**Results:** *Median age of the patients was 55 (44-67), with a male predomi- nance of 55%. Approximately 30% resided in a city other than the one in which the physician offering telemedicine was situated. Seventy-nine (16.7%) individuals received telemedicine via VC sessions without the requirement for any in-person examinations. The most prevalent disease among those who applied for telemedicine was asthma. Median duration of the initial VC session was 13 (8-18) minutes. At least half of the individuals seeking videoconfer- ence-based telemedicine for chronic respiratory disorders, such as asthma, COPD, and interstitial lung disease, had previously been followed by either the telemedicine provider or another physician in the same hospital. However, the vast majority of telemedicine applications in disease categories such as COVID, post-COVID, pulmonary nodules, and lung cancer were submitted by first-time applicants.
*

**Conclusion:** *This pioneering study suggests that videoconference-based telemedicine may be an alternative/complementary tool for patients, particularly those with chronic respiratory diseases.
*

**Key words:** *Outpatient clinic; respiratory diseases; telemedicine; videoconference; telepulmonology
*

**ÖZ**

**
Göğüs hastalıkları polikliniğinde videokonferansa dayalı teletıp deneyimi
**

**Giriş:** *Teletıp tüm dünyada, farklı alanlarda hızla yaygınlaşmaktadır. Bulaş riskinden korunma gerekliliği ve kronik hastaların takibinin çoğunlukta olması nedeniyle solunum yolu hastalıklarında kullanılabileceği bildirilse de teletıp deneyimine ilişkin yayınlar sınırlıdır. Bu çalışmanın amacı, teletıbbın göğüs hastalıkları kliniğinde uygun hastalarda kullanılmasına zemin hazırlamak amacıyla, video konferans tabanlı teletıp yoluyla değerlendirilen hastaların tanı, takip ve tedavi süreçlerinin retrospektif incelemesini yapmaktı.
*

**Materyal ve Metod:** *Tanımlayıcı nitelikteki bu çalışmada, Haziran 2020’den Şubat 2021’e sekiz ayda video konferans tabanlı teletıp ile değerlendirilen 478 hastanın tamamının anonimleştirilmiş tıbbi kayıtları incelendi. Demografik özellikler, hastalık öyküsü, ilk video konferans (VC) oturumu sonrasında fizik muayene/yüz yüze muayene gerekliliği, tetkiklerin yapılıp yapılmaması, ön tanı ve tanı süreçleri, takip süresi ve sonuçları analiz edildi. Haziran 2021’in sonunda çalışmaya katılan tüm hastaların takip verileri gözden geçi- rildi.
*

**Bulgular:** *Hastaların ortanca yaşı 55 (44-67) olup, %55 oranında erkek çoğunluktaydı. Yaklaşık %30’u teletıp sunan doktorun bulun- duğu şehirden farklı bir şehirde ikamet ediyordu. Yetmiş dokuz (%16,7) kişi yüz yüze muayeneye gerek kalmadan VC oturumları ile izlendi. En sık görülen hastalık astım idi. İlk VC oturumunun ortalama süresi 13 (8-18) dakikaydı. Astım, KOAH ve interstisyel akciğer hastalığı gibi kronik solunum bozuklukları için video konferansa dayalı teletıp ile değerlendirilen hastaların en az yarısı daha önce teletıp sağlayıcısı veya aynı hastanedeki başka bir göğüs hastalıkları hekimi tarafından takip edilmişti. Ancak COVID, post-COVID, akciğer nodülleri ve akciğer kanseri gibi hastalık kategorilerindeki teletıp başvurularının büyük çoğunluğu ilk kez başvuran hastalar tarafından yapıldı.
*

**Sonuç:** *Bu öncü çalışma, video konferans tabanlı teletıp uygulamasının özellikle kronik solunum yolu hastalıkları olan hastalar için alternatif/tamamlayıcı bir araç olabileceğini düşündürmektedir.
*

**Anahtar kelimeler:** *Göğüs hastalıkları; poliklinik; telepulmonoloji; teletıp; videokonferans
*

## INTRODUCTION

Telemedicine has gained popularity recently as a means of reducing the risk of cross-contamination during the COVID-19 pandemic although it is not a novel method for delivering healthcare support and has been studied for decades. Despite the method being known for a long time, the catalytic effect of the pandemic has required the widespread and varied implementation of telemedicine. World Health Organization (WHO) declared, quite a while ago, that the elements of telemedicine aim to provide clinical support, overcome geographical barriers, and use various techniques with the overall goal of improving health outcomes (1). Virtual visits are interactions between a patient and a healthcare professional through video, telephone, or live chat. Synchronous (or interactive real-time) telemedicine methods may be a substitute for traditional patient medical interviews. It has been shown that video conferencing is effective in delivering online treatment and is well-accepted by patients, as it simulated in-person, face-to-face consultation (2). Smith and colleagues have mentioned that telemedicine is an alternative for convenient access to routine care, avoiding the risk of exposure in crowded hospitals

and waiting areas during the pandemic period (3). A review of the literature on four diverse, prototypical medical conditions (stroke, diabetes, heart failure, and pregnancy) indicates that telemedicine is a safe and suitable alternative to traditional in-person models of care. These medical conditions span acute versus chronic as well as primary versus specialty care (4). Although many of the issues in respiratory medicine may cover the same features, the data in this field remain lacking.

Patients with respiratory diseases had to be those who should have an alternative for convenient access to routine care without facing the risk of COVID-19, considering that pulmonologists took a pivotal role in the management of COVID-19 patients. However, to date, there is no publication on the diagnostic variety of the patients applied to respiratory diseases outpatient clinics and managed by using telemedicine during the recent pandemic. Interestingly, Pacht et al. have published about the effectiveness of telemedicine in pulmonary outpatient clinics contemporaneously with the first WHO definition. The authors have found favorable results for telemedicine effectiveness in their prospective, crossover study, determining if there was any difference between care delivered

using video conferencing-based telemedicine technology and that given by a traditional face-to- face encounter in a pulmonary medicine clinic (5). Since telemedicine requires specific guidelines and recommendations for its development, the analysis of the data from the units where telemedicine is implemented as a routine way to provide healthcare in pulmonary medicine is crucial.

The most common diseases seen in that study (chronic obstructive pulmonary disease and asthma) and others like lung cancer, pulmonary fibrosis, and tuberculosis (even in a few numbers) might give a perspective on the scope of videoconference-based telemedicine in respiratory diseases. The purpose of this study was to conduct a retrospective evaluation of the prospective management of patients with respiratory diseases via videoconference-based telemedicine to lay the groundwork for its use in appropriate cases of pulmonology.

## MATERIALS and METHODS

Although all outpatient clinics in the University Hospital were reopened for traditional face-to-face visits after the first three months of the COVID-19 pandemic, a pulmonologist voluntarily transformed routine work on medical interviews with patients into telemedicine via VC session. The process of implementing this individually provided initiator system consists of an online appointment system based on an official website and the physician’s account on a VC program (Skype™, version 6.4, Microsoft, Redmond, WA, USA). The patients had appointments via the official website of University Hospital and a personalized meeting link had been sent to a smart phone number given by the patient at application. Whole video conferencing process had been described to applicants online before and during the attempt to get a remote VC appointment from the website. It is also described in a previous study (6). All patients who had an appointment system and attended at least one VC session for an eight-month period from June 1, 2020, to February 1, 2021, were included in the study.

For this observational descriptive study, the researchers retrieved the medical records of all patients from the detailed medical records of each anonymized patient. The medical notes were those kept by the pulmonologist on the personal computer and the hospital records of the officially registered patients. The parameters analyzed included

demographic characteristics (age, sex, place of residence, occupation, and marital status), disease history (newly diagnosed or previously diagnosed respiratory diseases and comorbidities), and information about how the patient attended the medical interview. Additionally, parameters such as the need for face-to-face physical examination after the first VC session, the inclusion of investigations, pre-diagnosis, diagnosis processes, follow-up period, and outcomes were analyzed. Follow-up data were reviewed for all patients enrolled in the study at the end of June 2021.

Follow-up evaluations were analyzed in three groups: ‘with only face-to-face controls,’ ‘with only online controls by video conferencing (including meetings for physical examination in some cases),’ and ‘hybrid controls (sometimes online controls, sometimes face- to-face controls).’ The time and duration for each online VC meeting were retrieved from the historical section of the video conferencing program.

Statistical analyses were conducted using SPSS 26.0 software (IBM SPSS Statistics Data Editor, Armonk, NY: IBM Corp.). Descriptive analysis was performed for all demographic features. All continuous variables were presented as medians with interquartile range (IQR) 25-75, and minimum-maximum values were provided when none of them followed a normal distribution. Categorical variables were reported as numbers and percentages. Chi-square test was employed to analyze differences in categorical groups. For variables assuming abnormal distribution, the Mann-Whitney U test was used for two-group comparisons and the Kruskal-Wallis test for more than two groups. A p-value of 0.05 was considered statistically significant.

This study was carried out in accordance with the Helsinki Declaration and was approved by the Ethical Council of the Pamukkale University in which this study was performed (Decision no: 03, Date: 02.02.2021).

## RESULTS

Between June 1, 2020 and February 1, 2021, the same pulmonologist conducted telemedicine via VC sessions for a total of 478 patients. Median age of the patients was 55 (44-67), with a male predominance of 55%. Approximately 30% resided in a city other than the one in which the physician providing telemedicine was located. Table 1 summarizes the sociodemographic features of the patients.

**Table:** 

Table 1. Sociodemographic features of the patients	
n (%)	
Sex	
Female	215 (45.0)
Male	263 (55.0)
Age (years) median (p25-p75)	55 (44-67)
Age groups	
≤45	133 (27.8)
46-64	200 (41.8)
65-84	141 (29.5)
≥85	4 (0.8)
Settlement	
In the city where the University Hospital located	343 (71.8)
In cities from same geographical region	106 (22.2)
In cities from other geographical regions	28 (5.8)
In cities from different country	1 (0.2)
Occupation	
Jobs not needed undergraduate education	224 (46.9)
Jobs needed undergraduate education	141 (29.5)
Housewife	73 (15.3)
Healthcare workers	24 (5.0)
Student	16 (3.3)
Working status	
Active working	216 (45.2)
Retired	99 (20.7)
Unemployed/Disabled/Housewife/Student	163 (34.1)

Out of the 79 (16.7%) individuals who received telemedicine via VC sessions without the need for

face-to-face physical examinations, only the first VC session was sufficient for 48 patients (17 of whom required counseling for COVID-related problems). The remaining 31 patients were followed up solely through VC sessions without the need to be physically present at the hospital. Apart from these patients, physical examinations were performed on all 399 patients.

During the initial VC session, inspection findings (such as clubbing, VCSS signs, cachexia, dermatological lesions on the leg, etc.) were recorded in 25 patients (5.2%). A total of 221 patients were invited to the hospital for a physical examination on the same day as the initial VC session while the rest were examined on another day after the first VC session, typically on their scheduled appointment day for further investigations. The flowchart of the study, outlining the patients’ evaluation methods, is summarized in Figure 1.

In terms of patient approach, 143 (29.9%) patients directly contacted the physician for the first VC session, 27 (5.6%) patients sought assistance from their relatives, and 55 (11.5%) patients were referred by another physician. Additional details are summarized in Table 2. All patients, except for 79 (16.5%), had reports of investigations conducted for their complaints before the first VC session. Records of these previous investigations in the national health system (e-Nabız) were available for 56 (11.7%) patients.

**Table:** 

Table 2. Features of the patients about attendance noted in the first videoconference (VC) session		
n (%)		
Attendance		
First attendance	143	(29.9)
Previously followed up by the University Hospital	95	(19.9)
Previously followed up by the physician who made telemedicine by videoconference (VC) sessions	147	(30.8)
A relative of the patient contacted with the physician beforehand	27	(5.6)
Referred by another physician	55	(11.5)
A family member of known healthcare workers	11	(2.3)
Investigation reports in first VC session		
None	79	(16.5)
Having the records about their disease in the hospital	234	(49.0)
Having national health system records (e-Nabız)	56	(11.7)
Keeping the reports on their own	109	(22.8)
Having previously diagnosed disease before first VC session		
No	86	(18.0)
Yes	392	(82.0)
Attended for the previously diagnosed disease	249	(52.0)
Attended for a new undiagnosed disease/disorder	143	(30.0)

**Table:** 

Table 3. Features of patients, first videoconference (VC) session and follow-up visits by disease categories										
									Pulmonary	
							Pulmonary	Pulmonary	vascular	Benign pleural
	Asthma	ILD	Post-COVID Malignancy		COPD	COVID-19	infection	nodule	disease	disease
	n= 133,	n= 94,	"n= 72,	n= 55,"		n= 47,	n= 36,	n= 19,	n= 18,	n= 12,	n= 6,
	n (%)	n (%)	"n (%)	n (%)"		n (%)	n (%)	n (%)	n (%)	n (%)	n (%)
Age (years)	49	60.5	52	67	67	50.5	58	53	72.5	58
Median (P25-P75)	(37-58)	(51-69)	(45.25-64.75)	(61-71)	(41-72)	(41-59.75)	(37-66)	(43.25-60.50)	(55.50-77.75)	(42-63)
Sex										
Male	50 (37.6)	56 (59.6)	38 (52.8)	45 (81.8)	35 (74.5)	23 (63.9)	10 (52.6)	12 (66.7)	4 (33.3)	3 (50.0)
Female	83 (62.4)	38 (40.4)	34 (47.2)	10 (18.2)	12 (25.5)	13 (36.1)	9 (47.4)	6 (33.3)	8 (66.7)	3 (50.0)
Attendance										
Previously followed up by the University Hospital	20 (14.9)	12 (12.8)	23 (31.9)	14 (25.5)	16 (34.0)	4 (11.1)	4 (21.1)	3 (16.7)	5 (41.7)	0 (0)
Previously followed up by the physician who	57 (42.8)	43 (45.7)	8 (11.1)	12 (21.8)	16 (34.0)	6 (16.7)	5 (26.3)	4 (22.2)	1 ( 3.3)	3 (50.0)
made telemedicine by VC sessions										
First attendance	56 (41.7)	39 (41.4)	41 (56.9)	29 (52.7)	15 (32.0)	26 (72.2)	10 (52.6)	11 (61.1)	6 (50.0)	3 (50.0)
Duration of first VC										
≤13 min	95 (71.4)	54 (57.4)	22 (30.6)	17 (30.9)	30 (63.8)	17 (47.2)	10 (52.6)	7 (38.9)	4 (33.3)	2 (33.3)
>13 min	38 (28.6)	40 (42.6)	50 (69.4)	38 (69.1)	17 (36.2)	19 (52.8)	9 (47.4)	11 (61.1)	8 (66.7)	4 (66.7)
Number of follow-up visits Median (P25-P75)										
Online	2(1-4)	3 (1-5)	3 (1-5)	2 (1-4)	1.5 (1-3.75)	3 (2-4)	4 (2-8)	2 (1-3)	1 (1-3.50)	2,50 (1.25-3.75)
Face-to-face	1 (1-2)	1 (1-2)	1 (1-2)	1 (1-2)	1 (1-2)	2 (1-2)	1 (1-2.50)	1 (1-2)	1 (1-2.25)	2 (1-3)
The median value of the percentage ratio of	100	71.4	"90.1	50"		88.8	83.3	73.3	100	45	83.3
VC controls among all controls* (P25-P75)	(72.7-100)	(50-100)	(50-100)	(0-100)	(83.3-100)	(60-100)	(55.3-100)	(80-100)	(0-100)	(54.1-100)
COPD: Chronic obstructive pulmonary disease, ILD: Interstitial lung disease, VC: Videoconference.										
										
*The number of online controls/the number of all controls.										


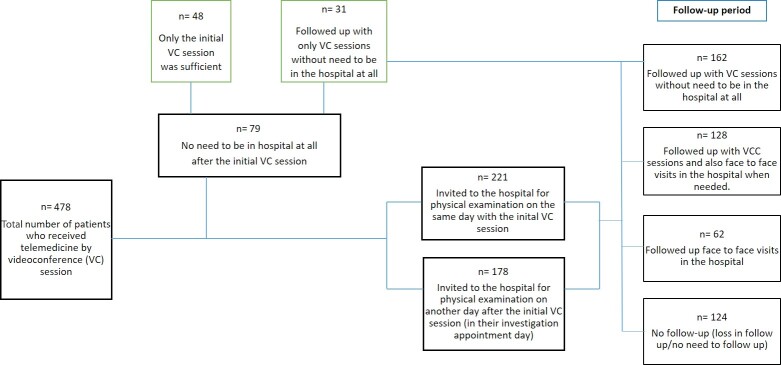
** Figure 1.** Study flow chart.

**Figure 1.** The flowchart of the study, outlining the patients' evaluation methods as in-person or videoconference (VC) session.

However, further investigations were sought after the first VC sessions in 52% (n= 269) of all patients, and this percentage increased to 68.3% (136/199) in patients with a previously undiagnosed condition. The total number of patients with previously diagnosed diseases was 392 (82.0%), but 143 (36.4%) of them consulted the physician for new undiagnosed diseases/disorders. Therefore, 47.9% (229/478) of the patients required further investigations to receive a new diagnosis while the rest were previously diagnosed patients with chronic respiratory diseases (Table 2). Among the previously diagnosed patient group, patients with asthma (n= 133) were the most frequent applicants.

After registering patients with multiple lung diseases in both illness categories separately, it was seen that the most prevalent disease among those who applied for telemedicine was asthma. Median duration of the initial VC session in all was 13 (8-18) minutes, but when disease groups were assessed, the pleural diseases category had the longest median duration (Table 3). Most patients had their first VC session completed in less than 13 minutes, particularly those with asthma and chronic obstructive pulmonary disease (COPD) (Table 3). At least half of those seeking telemedicine through videoconferencing for chronic lung disorders such as asthma, COPD, and

interstitial lung disease (ILD) had previously been followed by either the physician providing telemedicine or another physician in the same hospital. However, the majority of applications for telemedicine in disease categories such as COVID- 19, post-COVID complaints, pulmonary nodules, and lung cancer were generally first-time applicants (Table 3).

Within the patient population undergoing telemedicine, there were a total of 55 individuals with either previously diagnosed or newly diagnosed malignancies. A pathological diagnosis procedure for malignancy was planned for 32 patients. One patient did not attend the hospital, two were diagnosed with benign tumors, one patient passed away without a diagnosis, and four patients remained undiagnosed during the follow-up period. Among the remaining 24 patients, median time to diagnosis was 20 days (ranging from nine to 69 days).

Twenty-six patients were admitted to the hospital following the first VC session and/or physical examination, and an additional 28 patients were admitted during the follow-up period. Two patients were admitted twice: once following the first VC session and once following discharge with new indications. Hospitalizations occurred at a rate of 11.3%.

Median number of all controls conducted over the follow-up period, noted until February 1, 2021, was two (ranging from one to 22). Meanwhile, median number of face-to-face controls needed to supplement online controls was one (ranging from one to 12). Median frequency of online controls by videoconferencing, expressed as percentage [(number of online controls/number of all controls) x 100], was 83.3%. These percentage ratios of online controls by videoconferencing, categorized by disease, are presented in Table 3.

Up until June 30, 2021, data indicated that 93

(19.5%) patients fully recovered, 197 (41.2%) were still under observation, and 56 (11.7%) were determined to be out of follow-up. Twenty-two (4.6%) patients sought counseling alone (for a second opinion or due to concerns about their symptoms, especially in COVID-19 patients), 30 (6.3%) patients did not have a respiratory disease, and 20 (4.1%) patients were referred to another department or hospital. Unfortunately, 49 (10.3%) patients passed away; 26 of them had malignancies, six patients died due to COVID-19, five patients had interstitial lung disease (ILD), and the rest succumbed to miscellaneous reasons.

## DISCUSSION

This study presents the management of patients with respiratory diseases over an eight-month period, involving both VC sessions and in-person physical examinations when necessary. More than half of the studied population had previously been diagnosed with respiratory diseases. This groundbreaking study suggests that videoconference-based telemedicine could be a viable alternative tool for patients, especially those with chronic respiratory diseases, during the pandemic.

A study conducted in our country has demonstrated that, during the pandemic period, there was an 84% decrease in outpatient admissions and a 43% decrease in inpatients for respiratory diseases (7). However, patients with chronic diseases require continuous care and should not have been overlooked, even during the COVID-19 pandemic. Monitoring these patients consistently is essential to prevent acute symptoms related to their chronic diseases and disease progression. Continuous care for patients with chronic diseases during the pandemic is crucial because COVID-19-positive patients with chronic diseases are at significant risk

for infection and poor outcomes, including mortality (8-10). Sayani et al. have conducted a study addressing the cost and time barriers in chronic disease management through telemedicine. They found that telemedicine is economically beneficial, not only by reducing the socioeconomic barriers to cost and access but also by increasing the uptake of services (11).

The chronic lower respiratory disease (CLRD) group comprised 37.6% of the patient group who sought telemedicine during the study period. CLRD encompasses a group of disorders characterized by progressive or reversible airflow obstruction. Asthma and chronic obstructive pulmonary disorders (COPD), including chronic bronchitis and emphysema, are the two principal diseases included in CLRD (12). Given that many of these diseases have stable and exacerbation phases, medical supervision based on step-by-step techniques is indicated in follow-ups, or early detection of deterioration significantly improves disease progression (13,14). In the early period of the pandemic, McGee et al. published recommendations on telehealth services for people with CLRD and their informal caregivers. Despite being in a higher risk group for COVID-19, the authors noted that these patients and those caring for them constitute an underserved community due to pandemic measures (15).

In an umbrella review by Eze et al. (16), four investigations have explored the effectiveness of telemedicine for asthma management (first three) and COPD (the latest). Two reviews have found telemedicine to be at least as successful as face-to- face care, whereas two reviews have remained uncertain about telemedicine’s efficacy. According to the findings of the first evaluation, mobile app-based remote monitoring interventions that facilitated professional help improved asthma control and reduced exacerbation rates (17). The second review reported no difference in asthma symptom scores between the intervention groups (remote monitoring and telephone consults) and the groups receiving face-to-face care (18). The absence of a difference also supports telemedicine. The third review concluded that remote monitoring interventions had small beneficial effects on asthma control (19). In a study published at the beginning of the pandemic, the follow-ups of 328 asthma patients were managed using remote communication channels, and the researchers found no statistically significant difference

when comparing the current data of the participants with face-to-face data before follow-up with telemedicine (20). In our study, median value of the percentage ratio of VC controls among all follow-up controls in asthmatic patients was found to be 100%. This result may indicate that face-to-face interviews are needed less frequently in asthma patients, and they can be followed using VC sessions.

Median value of the percentage ratio of online controls by videoconferencing among all follow-up controls was 88.8% (83.3-100) in COPD patients. In the study of Cruz et al., they found limited evidence of the effect of home-based remote monitoring on reducing health care utilization, quality of life, and respiratory exacerbations in COPD patients (21). For the diagnosis and prognosis of COPD, performing an in-person spirometry examination is still required although telemedicine seems appropriate also for COPD follow-up. Home-based spirometry that is already in use for measuring forced vital capacity (FVC) parameter in different diseases needs to be studied to be added as a routine evaluation for COPD practice of telemedicine (22). Further studies and technologies are needed to stratify which patients, in terms of severity, will be best suited to a telemedicine management approach. Another area of potential growth is using artificial intelligence (AI) algorithms to determine developing COPD exacerbations (23).

A study on patient and clinician experience with telemedicine has found that “virtual video visits”, like the follow-up VC sessions in our study, may provide more effective and convenient follow-up than traditional in-person visits (24). The ideal process of medical history taking, which remains essential for accurate diagnosis, is described as either free of time limitations or with patient-centered features. This can be a real advantage of telemedicine, especially in extraordinary times. Lichstein reveals that the clinical hypotheses generated during the medical interview can help focus on physical examination and establish a basis for cost-effective utilization of diagnostic tests (25). Mackowiak discusses the value of physical contact via “touch” in favor of physical examination, using Reilly’s statistics indicating that nearly one in four inpatients had potentially care-altering physical examination findings (26,27). However, it also means that for the rest of the inpatients, the physical examination did not play such an essential role. Mackowiak also cites the relative contributions of

history, physical examination, and test results in making diagnoses as 60-80%, 10-20%, and 10-20%, respectively (26). To insist on ideal medical history taking that has retained its validity for decades, doctors need safe, comfortable, unrestricted conditions for interviews, just like all other interpersonal communications. The lack of immediate physical examination might be compensated for by frequent follow-up visits, which could also be among the advantages of videoconferencing (28). Adapting home-based remote monitoring interventions to patient characteristics and needs, the relationship and communication between the patient and healthcare professionals, and the usability and quality of the technology are factors that facilitate telemedicine (29).

Certain populations, such as single parents, immunocompromised patients, and those reliant on others for transportation, faced challenges during limited healthcare facility visitation, stay-home orders, and quarantine. Telemedicine can enhance access to health services, especially for individuals who depend on others for transportation (30). The age group most susceptible to contracting COVID-19 is those older than 65, with the highest mortality rate seen in those 85 and beyond (31). In the current study, the data supporting the utility of telemedicine pertain to this specific population. The median age of our study population was 55 (44-67), and one-third of our patients were over 65, with four patients over

85. In very elderly patients, videoconferencing was also used to connect with the family members of these patients in an effort to avoid further impairment in their health situation and provide comfort. In 2018, over one-fifth of Europe’s population was over the age of 65 (32). While the proportion of the elderly population in our country’s total population is 8.0%, in 2019, this rate increased to 9.1%. In the last five years, the elderly population has grown by 21.9%. It was determined that 23.5% of households had at least one elderly person (33). An aging population has placed significant pressure on public expenditures; consequently, telemedicine can enhance the size and efficiency of older patient delivery and continuous management. However, self-efficacy and digital literacy presumably have a significant impact on the uptake of telehealth among the elderly. In 5.6% of our study population, a relative of the patient contacted the physician before the first VC session personally with the patient, and the median age was 72 in this group. The capacity to reach the majority

of patients and caregivers is a unique benefit of portable devices like cell phones. Extensive use of mobile technologies makes outpatient and inpatient medical care more efficient, faster, safer, and less expensive (34).

Recently, Althobiani et al. have published the results of an international survey study on telehealth for interstitial lung diseases (ILD). While 38% of the 207 respondents declared using telehealth for managing ILD patients remotely, they admitted to using it for monitoring disease progression (70%), quality of life (63%), medication use (63%), and reducing the need for in-person visits (63%) (35). The progression of the disease and its severe negative effects on health quality underscore the vital role of regularly monitoring ILD patients. On the other hand, the importance of taking a detailed medical history, including exposures, drugs, habits, and accompanying underdiagnosed diseases, necessitates a lengthy interview time, which is facilitated and made comfortable via telemedicine in pandemic conditions. The high number of ILD patients in the current study is related to the physician’s expertise in that field, being in a tertiary referral center. Additionally, most of the ILD patients were individuals who had already been treated and followed by the same clinician. For patients who had undergone previous testing for the differential diagnosis of ILD, the evaluation of results remotely from personal health data systems, making treatment decisions in a patient-centered manner, and planning follow-ups can be easily performed during VC sessions. For first-time applicants, diagnostic tools might be organized according to the guidelines in the light of clinical clues. On the other hand, home monitoring in ILD patients via technological tools such as home-based spirometry, pulse oximeter, cough detector, etc., has been discussed comprehensively recently (36).

Lung cancer, the most prevalent and lethal cancer, significantly contributes to outpatient applications for respiratory diseases. Early detection of lung cancer is crucial for improving survival rates (37). A study examining the time taken from the onset of symptoms to the diagnosis of lung cancer in regular outpatient clinics has found a median diagnosis time of 49 days, varying between 12 and 396 days (38). In a comprehensive analysis of delays in the diagnosis and treatment of lung cancer, the impact of difficulty in reaching physicians on diagnostic delays was emphasized (39). Another study on delays in diagnosis

and treatment identified delays in counseling investigations and insufficient time for patient evaluation as contributing factors to delayed diagnosis (40).

In our study, median time for diagnosing lung cancer was 20 days (ranging from nine to 69 days) for 32 patients following a diagnostic procedure planned at the end of the first VC session. This result suggests that the use of videoconference-based telemedicine in diagnosing lung cancer does not prolong the diagnostic timeline. Further research is needed to determine if videoconference-based telemedicine can effectively address challenges in accessing healthcare, delays in counseling investigations, and insufficient time for patient evaluation, as identified in previous studies on this topic.

Evaluating qualitative data to assess patient perspectives from those receiving videoconference- based telemedicine by the same physician can complement the quantitative outcomes of the current study. While these patients may not be part of the same sample, their feedback may highlight certain advantages of this approach, such as avoiding the risk of contagion from hospital exposure and saving time and money by consulting the physician online when necessary (41). Considering patients’ experiences played a crucial role in enhancing the holistic aspects of this voluntarily adopted telemedicine model. Future studies should concentrate on assessing the outcomes of VC sessions for each disease individually and comparing the effectiveness of traditional and telemedicine methods employed by the same physician.

In a pre-pandemic review, it was revealed that asthma, COPD, neuromuscular diseases, ventilator- assisted individuals, and patients on pulmonary rehabilitation programs may benefit from telemedicine (42). More recently, it has been suggested that virtual visits offer a significant opportunity to improve care quality, connectivity, efficiency, and equity. Moreover, the importance of maintaining web-based visits beyond the pandemic has been emphasized as an opportunity for high-quality, patient-centered healthcare (43). The advantages noted by healthcare providers and patients could serve as a compelling reason to persist with telemedicine through video conferencing method, regardless of extraordinary conditions. Real-life experiences’ results would contribute to boosting confidence.

## CONCLUSION

In conclusion, looking ahead to the future of telemedicine in pulmonary medicine, technological tools such as wearables, smart inhalers, portable electronic spirometers, digital stethoscopes, and clinical decision support systems may help pulmonologists enhance the quality of care, improve therapy adherence, and enable early detection of worsening in chronic pulmonary diseases (44). With advancements in reimbursement and legal regulations, adherence to ethical and licensing principles, and improvements in technological infrastructure, telemedicine may become a routinely used strategy for diagnosis and follow-up in acute and chronic respiratory diseases.

**Ethical Committee Approval:** This study was approved by the Pamukkale University Non-Invasive Clinical Research Ethics Committee (Decision no: 03, Date: 02.02.2021).

## CONFLICT of INTEREST

The authors declare that they have no conflict of interest.

## AUTHORSHIP CONTRIBUTIONS

Concept/Design: NC, PB, GA Analysis/Interpretation: NC, PB Data acqusition: NC, PB, GA Writing: NC, PB, GA

Clinical Revision: NC, PB, GA Final Approval: NC, PB, GA

## REFERENCES

1. *
  World Health Organization (WHO). A health telematics policy in support of WHO’s health-for-all strategy for glob- al health development: Report of the WHO group consul- tation on health telematics, 11-16 December, Geneva, 1997. Available from: https://apps.who.int/iris/han- dle/10665/63857 (Accessed date: 15.11.2023).
  *
2. *
  Almathami HKY, Win KT, Vlahu-Gjorgievska E. Barriers and facilitators that influence telemedicine-based, real-time, online consultation at patients’ homes: Systematic litera- ture review. J Med Internet Res 2020; 22(2): e16407. https://doi.org/10.2196/16407*
3. *
  Smith AC, Thomas E, Snoswell CL, Haydon H, Mehrotra A, Clemensen J, et al. Telehealth for global emergencies: Implications for coronavirus disease 2019 (COVID-19). J Telemed Telecare 2020; 26(5): 309-13. https://doi. org/10.1177/1357633X20916567*
4. *
  Barbosa W, Zhou K, Waddell E, Myers T, Dorsey ER. Improving access to care: Telemedicine across medical domains. Annu Rev Public Health 2021; 42: 463-81. h t t p s : / / d o i . o r g / 1 0 . 1 1 4 6 / a n n u r e v - publhealth-090519-093711*
5. *
  Pacht ER, Turner JW, Gailiun M, Violi LA, Ralston D, Mekhjian HS, et al. Effectiveness of telemedicine in the outpatient pulmonary clinic. Telemed J 1998; 4(4): 287- 92 . https://doi.org/10.1089/tmj.1.1998.4.287*
6. *
  Bostan P, Çetin N, Altınışık G. A retrospective assessment of the continuous health care provided to COVID-19 patients consulted via videoconference. Thorac Res Pract 2023; 24(1): 14-21. https://doi.org/10.5152/ ThoracResPract.2023.22058*
7. *
  Sevinç C, Tertemiz KC, Atik M, Güler N, Ulusoy M, Coşkun F, et al. How were non-COVID pulmonary patients and diseases affected from COVID-19 pandemic period? Turk Thorac J 2021; 22(2): 149-53. https://doi.org/10.5152/ TurkThoracJ.2021.20249*
8. *
  Klompas M. Coronavirus disease 2019 (COVID-19): Protecting hospitals from the invisible. Ann Int Med 2020; 172: 619-20. https://doi.org/10.7326/M20-0751*
9. *
  Bhaskar S, Rastogi A, Chattu VK, Adisesh A, Thomas P, Alvarado N, et al. Key strategies for clinical management and improvement of healthcare services for cardiovascu- lar disease and diabetes patients in the coronavirus (COVID-19) settings: Recommendations from the REPROGRAM consortium. Front Cardiovasc Med 2020; 7: 112. https://doi.org/10.3389/fcvm.2020.00112*
10. *
  Bhaskar S, Bradley S, Israeli-Korn S, Menon B, Chattu VK, Thomas P, et al. Chronic neurology in COVID-19 era: Clinical considerations and recommendations from the REPROGRAM consortium. Front Neurol 2020; 11: 664. https://doi.org/10.3389/fneur.2020.00664*
11. *
  Sayani S, Muzammil M, Saleh K, Muqeet A, Zaidi F, Shaikh
  *

*
T. Addressing cost and time barriers in chronic disease management through telemedicine: An exploratory research in select low- and middle-income countries. Ther Adv Chronic Dis 2019; 10: 2040622319891587. https:// doi.org/10.1177/2040622319891587*

12. *
  World Health Organization. Chronic respiratory diseases. Available from: https://www.who.int/health-topics/chron- ic-respiratory-diseases#tab_tab_1 (Accessed date: 15.07.2023).
  *
13. *
  Global Initiative for Chronic Obstructive Lung Disease (GOLD). Global strategy for prevention, diagnosis and management of COPD: 2022 report. Available from: https://goldcopd.org/2022-gold-reports-2/
  *
14. *
  Global Initiative for Asthma (GINA). Global Strategy for Asthma Management and Prevention 2022 Report. Available from: https://ginasthma.org/wp-content/ uploads/2022/07/GINA-Main-Report-2022-FINAL-22-07- 01-WMS.pdf (Accessed date: 01.06.2022).
  *
15. *
  McGee JS, Meraz R, Myers DR, Davie MR. Telehealth services for persons with chronic lower respiratory disease and their informal caregivers in the context of the COVID- 19 pandemic. Practice Innovations 2020; 5(2): 165-77. https://doi.org/10.1037/pri0000122*
16. *
  Eze ND, Mateus C, Hashiguchi TCO. Telemedicine in the OECD: An umbrella review of clinical and cost-effective- ness, patient experience and implementation. PloS One 2020; 15(8): e0237585. https://doi.org/10.1371/journal. pone.0237585*
17. *
  Hui CY, Walton R, McKinstry B, Jackson T, Parker R, Pinnock H. The use of mobile applications to support self-management for people with asthma: A systematic review of controlled studies to identify features associated with clinical effectiveness and adherence. J Am Med Inform Assoc 2017; 24: 619-32. https://doi.org/10.1093/ jamia/ocw143*
18. *
  Zhao J, Zhai YK, Zhu WJ, Sun DX. Effectiveness of telemed- icine for controlling asthma symptoms: A systematic review and meta-analysis. Telemed J E Health 2015; 21: 484-92. https://doi.org/10.1089/tmj.2014.0119*
19. *
  McLean G, Murray E, Band R, Moffat KR, Hanlon P, Bruton
  *
  - *
    Interactive digital interventions to promote self-man- agement in adults with asthma: Systematic review and meta-analysis. BMC Pulm Med 2016; 16(1): 83. https:// doi.org/10.1186/s12890-016-0248-7*
20. *
  Simsek SM, Kizilirmak D, Havlucu Y. The effectiveness of telemedicine methods in the follow-up of asthma in the COVID-19 pandemic process. Eur Respir J 2021; 58: PA956.
  *
21. *
  Cruz J, Brooks D, Marques A. Home telemonitoring effec- tiveness in COPD: A systematic review. Int J Clin Pract 2014; 68: 369-78. https://doi.org/10.1111/ijcp.12345*
22. *
  Nathan SD, Wanger J, Zibrak JD, Wencel ML, Burg C, Stauffer JL. Using forced vital capacity (FVC) in the clinic to monitor patients with idiopathic pulmonary fibrosis (IPF): Pros and cons. Expert Rev Respir Med 2021; 15(2): 175-81. https://doi.org/10.1080/17476348.2020.18168 31*
23. *
  Blakey JD, Bender BG, Dima AL, Weinman J, Safioti G, Costello RW. Digital technologies and adherence in respi- ratory diseases: The road ahead. Eur Respir J 2018; 52: 1801147. https://doi.org/10.1183/13993003.01147- 2018*
24. *
  Donelan K, Barreto EA, Sossong S, Michael C, Estrada JJ, Cohen AB, et al. Patient and clinician experiences with telehealth for patient follow-up care. Am J Manag Care 2019; 25: 40-4.
  *
25. *
  Lichstein PR. The Medical Interview. In: Walker HK, Hall WD, Hurst JW, editors. Clinical Methods: The History, Physical, and Laboratory Examinations. 3rd ed. Boston:
  *

*
Butterworths; 1990. Chapter 3. PMID:21250190.
*

26. *
  Mackowiak PA. COVID-19, telemedicine, and the demise of the physican’s touch. South Med J 2021; 114(4): 237-8. https://doi.org/10.14423/SMJ.0000000000001229*
27. *
  Reilly BM. Physical examination in the care of medical inpatients: An observational study. Lancet 2003; 362(9390): 1100-5. https://doi.org/10.1016/S0140- 6736(03)14464-9*
28. *
  Hampton JR, Harrison MJ, Mitchell JR, Prichard JS, Seymour C. Relative contributions of history-taking, phys- ical examination, and laboratory investigation to diagnosis and management of medical outpatients. Br Med J 1975; 2(5969): 486-9. https://doi.org/10.1136/bmj.2.5969.486*
29. *
  Radhakrishnan K, Xie B, Berkley A, Kim M. Barriers and facilitators for sustainability of tele-homecare programs: A systematic review. Health Serv Res 2016; 51: 48-75. https://doi.org/10.1111/1475-6773.12327*
30. *
  Bhaskar S, Bradley S, Chattu VK, Adisesh A, Nurtazina A, Kyrykbayeva S, et al. Telemedicine as the new outpatient clinic gone digital: Position paper from the Pandemic Health System Resilience Program (REPROGRAM) International Consortium (Part 2). Front Public Health 2020; 8: 410. https://doi.org/10.3389/fpubh.2020.00410*
31. *
  CDC COVID-19 Response Team. Severe outcomes among patients with Coronavirus disease 2019 (COVID-19)- United States, February 12-March 16, 2020. MMWR Morb Mortal Wkly Rep 2020; 69(12): 343-6. https://doi. org/10.15585/mmwr.mm6912e2*
32. *
  European Commission. 2018 Ageing Report: Policy chal- lenges for ageing societies. Available from: https://ec.eu- ropa.eu/info/news/economy-ﬁnance/policy-implica- tions-ageing-examined-new-report-2018-may-25_en (Accessed date: 18.06.2022).
  *
33. *
  Turkish Statistical Institute. Available from: https://data. tuik.gov.tr/ (Accessed date: 03.06.2022).
  *
34. *
  Honeyman E, Ding H, Varnﬁeld M, Karunanithi M. Mobile health applications in cardiac care. Int Cardiol 2014; 6: 227-40. https://doi.org/10.2217/ica.14.4*
35. *
  Althobiani M, Alqahtani JS, Hurst JR, Russell AM, Porter J. Telehealth for patients with interstitial lung diseases (ILD): Results of an international survey of clinicians. BMJ Open Resp Res 2021; 8: e001088. https://doi.org/10.1136/ bmjresp-2021-001088*
36. *
  Wijsenbeek MS, Moor CC, Johannson KA, Jackson PD, Khor YH, Kondoh Y, et al. Home monitoring in interstitial lung diseases. Lancet Respir Med 2023; 11(1): 97-110. https://doi.org/10.1016/S2213-2600(22)00228-4*
37. *
  Verma A, Lim AYH, Tai DYH, Goh SK, Kor AC, A DBA, et al. Timeliness of diagnosing lung cancer: Number of pro- cedures and time needed to establish diagnosis: Being right the first time. Medicine (Baltimore) 2015; 94(29): e1216. https://doi.org/10.1097/MD.0000000000001216*
38. *
  Yaman N, Ozgen A, Celik P, Ozyurt BC, Nese N, Coskun AS, et al. Factors affecting the interval from diagnosis to treatment in patients with lung cancer. Tumori 2009; 95(6): 702-5.* *https://doi.* *org/10.1177/030089160909500611*
39. *
  Vinas F, Ben Hassen I, Jabot L, Monnet I, Chouaid C. Delays for diagnosis and treatment of lung cancers: A systematic review. Clin Respir J 2016; 10(3): 267-71. https://doi.org/10.1111/crj.12217*
40. *
  Yurdakul AS, Kocatürk C, Bayiz H, Gürsoy S, Bircan A, Özcan A, et al. Patient and physician delay in the diagno- sis and treatment of non-small cell lung cancer in Turkey. Cancer Epidemiol 2015; 39(2): 216-21. https://doi. org/10.1016/j.canep.2014.12.015*
41. *
  Altinisik Ergur G, Nuhoglu S, Cobanoglu C, Sengul M, Eryildiz N, Ergur A. The patient perspective of telemedi- cine in the context of COVID-19 pandemic. Bull Sci Technol Soc 2022; 42(1-2): 39-53. https://doi. org/10.1177/02704676221094735*
42. *
  Ambrosino N, Makhabah DN, Sutanto YS. Tele-medicine in respiratory diseases. Multidiscip Respir Med 2017; 12: 9. https://doi.org/10.1186/s40248-017-0090-7*
43. *
  Patel M, Berlin H, Rajkumar A, Krein SL, Miller R, DeVito J, et al. Barriers to telemedicine use: Qualitative analysis of provider perspectives during the COVID-19 pandemic. JMIR Hum Factors 2023; 10: e39249. https://doi. org/10.2196/39249*
44. *
  Honkoop P, Usmani O, Bonini M. The current and future role of technology in respiratory care. Pulm Ther 2022; 8(2): 167-79. https://doi.org/10.1007/s41030-022- 00191-y*

